# Life history traits variation in heterogeneous environment: The case of a freshwater snail resistance to pond drying

**DOI:** 10.1002/ece3.68

**Published:** 2012-01

**Authors:** Elodie Chapuis, Jean-Baptiste Ferdy

**Affiliations:** 1 UMR CBGP,Campus de Baillarguet, Montferrier-sur-Lez CedexFrance; 2 Évolution et Diversité Biologique (EDB),Université Paul SabatierFrance

**Keywords:** Common garden experiment, heterogeneous environments, life history traits, local adaptation, temporary habitats

## Abstract

Ecologists and population geneticists have long suspected that the diversity of living organisms was connected to the structure of their environment. In heterogeneous environments, diversifying selection combined to restricted gene flow may indeed lead to locally adapted populations. The freshwater snail, *Galba truncatula*, is a good model to address this question because it is present in a heterogeneous environment composed of temporary and permanent waters. In order to test the selective importance of those environments, we proposed here to measure survival of lineages from both habitats during drought episodes. To this purpose, we experimentally submitted adults and juveniles individuals from both habitats to drought. We found a difference in desiccation resistance between temporary and permanents waters only for adults. Adults from temporary habitats were found more resistant to drought. This divergence in desiccation resistance seems to explain the unexpected life history traits differences between habitats observed.

## Introduction

Selection in heterogeneous environments has been one of the mechanisms proposed by various theoretical studies to maintain genetic variation (e.g., Levene 1953; Dempster 1955 and reviews in Felsenstein 1976; Hedrick et al. 1976; Hedrick 2006). A spatially heterogeneous environment is a series of patches or habitats of various conditions, and diversifying selection between habitats may lead to local adaptation of individuals. This means that local genotypes would have a higher relative fitness in their habitat of origin than genotypes from other habitats (Williams 1966). Numerous theoretical studies have focused on the ecological conditions that permit local adaptations in heterogeneous habitats (e.g., Christiansen 1975, 1985; Hedrick 1990). In parallel, abundant empirical studies have also been conducted to detect local adaptation with William's definition (1966), using reciprocal transplant experiments on various animal and numerous plant species (reviews in Kawecki and Ebert 2004; Leimu and Fischer 2008). However, reciprocal transplant experiments are not always feasible for technical or/and ethical reasons. This is why other methods to study local adaptation in laboratory conditions have been developed (Kawecki and Ebert 2004). (1) The experimental evolution approach helped to explore relationships between selection, environment, and life history trait evolution (reviewed in Kassen 2002; Byers 2005) (2) The comparison of quantitative versus molecular differentiation between populations, that is, the *Q*_ST_–*F*_ST_ comparison, can infer the relative importance of drift and selection in phenotypic evolution (Merilä and Crnokrak 2001; McKay and Latta 2002). (3) At last, common garden experiments where, the environmental factor thought to be responsible for a selective pressure should be mimicked in laboratory condition, while other environmental factors are kept constant. It has been successfully used, for example, to test adaptation of a parasite to its host (Lively 1989; Thrall et al., 2002).

Freshwater species that colonize temporary and permanent waters may be good candidates for studies of life history traits evolution in heterogeneous environment. Indeed, their habitat is considered as spatially heterogeneous because it is composed of two distinct environment differing in water availability: temporary and permanent environments (Dillon 2000). These different environmental conditions have been shown to be the cause of population differentiation in several freshwater species (Wellborn et al. 1996), including freshwater snails (Appleton 1978; Jarne and Städler 1995). In those case studies, differences in life history traits can be related to differences in physical tolerance to drought, and thus drought can be considered as a potential selective agent (Williams 2006).

*Galba truncatula*, a freshwater snail, is one of these interesting system species that are present in both temporary and permanent habitats (Trouvé et al., 2003; Chapuis et al., 2007). In previous studies, we found no molecular genetic differentiation but strong differences for life history traits among habitats in *Galba truncatula*, that is, *Q_ST_* > *F_ST_* (Chapuis et al., 2007, 2008). This pattern supports the idea that diversifying selection leads to local adaptation between temporary and permanent waters and affect life history traits. But the *Q_ST_–F_ST_* comparison does not provide any clue on the selective force that drives local adaptation and phenotypic variation in the wild can be a biased estimation of the quantitative genetic variation (Pujol et al., 2008). This issue can be addressed by using an experimental approach. In the *Galba truncatula* environment, the obvious factor differing between the two environments is the high probability of desiccation events in the temporary habitat compared to permanent habitats (Kendall 1953; Goumghar et al. 2001; Trouvé et al. 2003; Ilg et al. 2011). It is therefore expected that selection should favor individuals that are the most resistant to drought in temporary habitat but not necessarily in permanent waters (Kendall 1953; Williams 2006). In this paper, we will test adaptation to temporary waters in laboratory conditions. For this purpose, we will measure drought tolerance of individuals that originate from both permanent and temporary individuals, with the expectation that the latter should be more resistant to drought if desiccation is indeed the mechanism that drives local adaptation. We will perform these measurements at two development stages (adult and juveniles) and control for traits, such as body size or age that are known to influence drought resistance (Dudgeon 1981; Facon et al. 2004).

## Materials and Methods

### Origin of individuals

**Figure 1:**
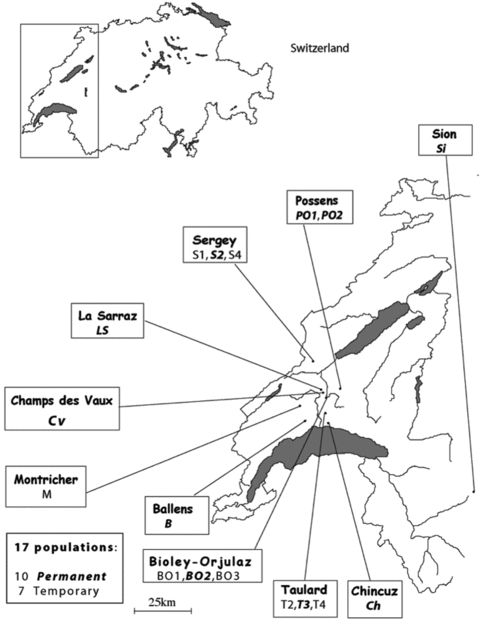
Sampling locations of the 17 populations studied in western Switzerland. Populations from permanent water habitat are represented in bold italics and those from temporary water habitat in normal font. The two habitat types can co-occur within the same locality and thus be few centimeters or meters apart.

Seventeen populations of *Galba truncatula* were sampled in Western Switzerland in spring 2003 (Fig. 1). Ten populations were coming from permanent habitat, and seven populations were coming from temporary habitats (for more details on populations and identification of habitats see Chapuis et al. 2007). Those populations from temporary and permanent habitats could be apart from only few meters or even centimeters (Chapuis et al. 2007; Fig.1). We know that those populations even close do not mix a lot because of a high *F*_ST_ values between pairs of populations found previously (Trouvé et al. 2005; Chapuis et al. 2007). We brought adults back from the field and, as these snails are preferential selfers, we kept them in individual Petri dish for every breeding generation, allowing only self-fertilization (Chapuis et al. 2007). Adults (i.e., sexually mature individuals) were issued from the second generation of laboratory breeding (G2), whereas juveniles (i.e., before sexual maturity) were issued from the third generation (G3). Tested individuals could not be issued from the same generation because desiccation test is lethal. Moreover, the use of the second and the third generations eliminates most of the potential maternal effects (Lynch and Walsh 1998). The first generation (G1) was used for previous studies comparing molecular and quantitative differentiation between populations and several life history traits were measured like size and age at maturity (Chapuis et al. 2007, 2008). Consequently, for the desiccation tests, we used 160 G2 adults (80 from temporary populations and 80 from permanent populations). Part of the G2 individuals was reared in fall, and then spent wintertime as juvenile before reproducing in spring. Other individuals were reared in spring and then laid egg rapidly. Age might impact body size and other physiological characteristics, but we know that age and size are strongly correlated (Chapuis et al. 2008). Thus, the estimation of rearing season (not age) will then be used in our statistical analysis. At last, we utilized 239 juveniles from the third generation (G3), laid before the drought experiment on adults, to run our desiccation tests on juveniles (99 individuals from temporary populations and 140 from permanent populations).

### Experimental design

#### Adults

G_2_ adults were submitted to the desiccation treatment at the same physiological stage, that is, 31 days after they reached sexual maturity. Sexual maturity was determined when they laid their first egg clutch by selfing. Since sexual maturity varied between individuals, the desiccation treatment did not begin at the same time for every snail, the age at maturity is recorded. Adult sizes were obtained before the desiccation treatment by measuring the length of the shell with an ocular micrometer under a binocular microscope as in G_1_ (Chapuis et al., 2007). Individuals were then placed into Petri dishes of 5-cm diameter at a constant room temperature of 25°C, with 24 h of light and 80% of humidity. The desiccation treatment consisted in leaving individuals in those conditions without any supplementation of water. All individuals had their operculum faced to the bottom of the dishes since it has been observed that in natural conditions snails enter in aestivation with the shell aperture applied to the mud surface (Kendall 1949). After 7 days of treatment, individuals were put into water for 1 h to check for their survival, and alive individuals were then put back into the desiccation treatment. We checked for survival, in an identical way, four additional times, every 7 days (i.e., a total of five checking events over 5 weeks). We chose this timing of 7 days because it corresponds to the upper limit of ordinary water restriction in natural temporary ponds we sampled (E.C., personal observation). In the same manner, the 5 × 7 days period was chosen because it allowed well-represented differences between habitats resistance (E.C., personal observation).

#### Juveniles

A similar experimental design was performed on juveniles G_3_ from 13 populations over the 17 initial samples used in G2 (because we could not obtain G3 individuals from two of the permanent habitat populations and two of temporary habitat populations). Juveniles, all of them aged 35 days after hatching, were submitted to the desiccation treatment and their size was also measured like adults. Contrary to adults, survival was checked only 2 and 4 h after the beginning of the desiccation treatment. We chose this shorter time interval because in preliminary experiments, we observed almost a complete juvenile mortality after 4 h of desiccation (i.e., no variance in mortality between individuals after this time). Survival was checked as for adults, except that we kept juveniles into water for only 15 min, as the time required to recover active life when back into water seems to be correlated to the time spent under desiccation (E.C., personal observation).

### Statistical analyses

#### Comparison of phenotypic values among habitats and across generations

Differences in adult age and size (for adults and juveniles) between G2 individuals of both habitats were analyzed by two-tailed tests using R (R Development Core Team 2008). We then compared these results to those obtained with G1 individuals (Chapuis et al. 2007).

#### Survival analyses

We analyzed our data continuously using every survival checking point to have more statistical power. Our analyses were done in two steps, using nonparametric and parametric analyses. Nonparametric methods are robust and quite easy to apply to our data. Conversely, parametric methods are more powerful but require that the distribution of survival time is known.

First, the survival curve for each habitat was built by computing the Kaplan–Meier estimators (Kaplan and Meier 1958) using the package SURVIVAL (Therneau and Lumley 2008) developed for the statistical software R (R Development Core Team 2008). The Kaplan–Meier estimator is a nonparametric method that allows analyzing observations for which the complete distribution is not known (i.e., censored data). It computes survival at a given time point as the product of the conditional survival probabilities over all preceding time intervals. Then, the different survival distributions were compared with a log-rank test (also nonparametric).

At last, the effects of several factors on survival were tested. By a nonparametric method, with a general mixed-effects Cox model (Cox, 1972), using the function *coxme* in the package KINSHIP (Atkinson and Therneau 2008) in R. We could thus conduct tests with random factors. The effects of adult body size, rearing season, and habitat of origin as fixed effect, with populations as random effect, were tested. Then, we conducted a parametric analysis using survival models implemented in the *survreg* function in the package SURVIVAL in R (R Development Core Team 2008; Therneau and Lumley 2008) testing body size, adult rearing season, and habitat of origin. This analysis, unlike the Cox model, allowed us to choose among several possible distributions of time before death. For instance, an exponential distribution is a one-parameter distribution, which supposes that death rate is constant through time. However, in many cases the death rate may vary with time. We chose here to analyze our data with a Weibull distribution, this choice proved to maximize the likelihood of our statistical models, since we previously tested to all distributions. In this model, a scale parameter is fitted that describes how death rate changes through time. A scale of one indicates that death rate is constant; scales superior to one mean that mortality increases with time. Finally, a scale smaller than one indicates that death rate decreases over time. This latter situation should be expected if individuals differ in their resistance, because, as the least resistant individuals die first, average resistance increases over time. Inspecting this scale parameter is therefore a way to test for the existence of variation in resistance among individuals.

## Results

### Comparison of t phenotypic values among habitats and across generations

For juveniles, no size difference was detected between habitats but it was not surprising because in G_1_ no difference was noticed (Chapuis et al. 2007). Considering adults, no statistically significant differences between habitats for age and size between G_2_ individuals of both habitats (Table 1). But this time, we can be astonished: differences for both traits were found on G_1_ individuals (see Chapuis et al. 2007 and Table 1). One can argue that this discrepancy between G_1_ and G_2_ is due to maternal effects in G_1_ that had disappeared in G_2_, although we consider the occurrence of maternal effects in G_1_ quite unlikely (Chapuis et al. 2007). Another explanation of this inconsistency between G_1_ and G_2_ could be that we have lower statistical power for the G_2_, because of smaller sample sizes. To test for this second possible explanation, we ran Wilcoxon tests between temporary and permanent habitats by bootstrapping (3,000 times) G_1_ data with sample size as in G_2_. The value of the Wilcoxon statistics we obtained for G_2_ individuals falls within the 95% confidence interval of *W* values obtained by bootstrapping G_1_ data with sample size as in G_2_ (bootstrap *P* > 0.05).

**Table 1:** Phenotypic values (the standard error) of the age and the size at maturity for individuals from temporary (T) and permanent (P) habitats and for the two generations of breeding: G_1_ and G_2_. Sample size for each habitat per generation is given (e.g., NP is the sample size for the permanent habitat). G_2_ is splitted according the two seasons of rearing (i.e., fall and spring) The *P*-value of the two-tailed Wilcoxon test is given for each case and *P*-values in bold are significant at the 0.05 level

		G_1_ NP = 438; NT = 317		G_2_-all NP = 80; NT = 80		G_2_-spring NP = 39; NT = 50		G_2_-fall NP = 41; NT = 30	
					
Trait	Habitat	Mean	Wilcoxon-test	Mean	Wilcoxon-test	Mean	Wilcoxon-test	Mean	Wilcoxon-test
Adult	P	99.22		158.59		108.26		219.69	
age		(1.281)	***P*****< 0.01**	(7.62)	*P* = 0.76	(4.38)	*P* = 0.77	(4.61)	*P* = 0.18
(days)	T	111.88		145.77		112.83		210.5	
		(1.285)		(6.36)		(4.84)		(5.59)	
Adult	P	3.57		3.95		3.8		4.08	
*size*		(0.016)	***P*****< 0.01**	(0.046)	*P* = 0.46	(0.065)	*P* = 0.71	(0.061)	*P* = 0.07
(mm)	T	3.76		3.91		3.85		3.98	
		(0.019)		(0.032)		(0.040)		(0.053)	

This confirms that the lack of difference in adult size and age in the second generation is presumably due to small sample size. As G_1_ individuals were reared in spring, we were interested into values of size and age considering the rearing season. Doing so, we can see the difference between habitats remains nonsignificative because of sampling size (Table 1) but in the same way that in G_1_: temporary individuals mature later and at a bigger size than permanent ones.

### Habitat effect

**Figure 2:**
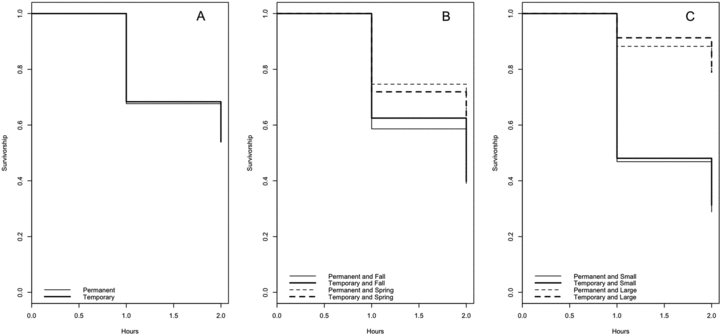
Kaplan–Meier survival curves for juveniles: the proportion of surviving individuals function of hours spent under the desiccation treatment. (A) Combined for both habitats: Permanent and Temporary. (B) For both habitats considering the rearing season: spring and fall. (C) For both habitats considering body size: small and large.

**Figure 3:**
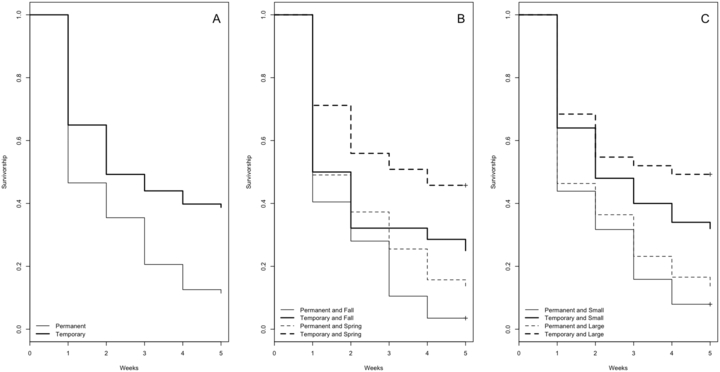
Kaplan–Meier survival curves for adults: the proportion of surviving individuals function of weeks spent under the desiccation treatment. (A) Combined for both habitats: Permanent and Temporary. (B) For both habitats considering the rearing season: spring and fall. (C) For both habitats considering body size: small and large.

Juveniles from both habitats showed no differences in survival rate during the two series of desiccation as illustrated on Kaplan–Meier figures (Fig. 2A). The Log rank analysis detected no habitat effect (data not shown). Conversely, we observed a large difference between survival curves of temporary and permanent habitats for adults (Fig. 3A). This difference is still detected when controlling for size and season on survival with the nonparametric analysis (Table 3) and also with the parametric analysis (Table 4A).

**Table 3:** Analysis of adult survival using a random effect nonparametric Cox model with the effect of adult body size, rearing season, habitat, and population as random effect. *P*-values in bold are significant at the 0.05 level

Source	Coefficient	Se (coef)	*P*
Temporary habitat	−0.544	0.255	0.062
Size	−0.741	0.284	**0.009**
Spring season	−0.555	0.205	**0.006**
Population			0.115

**Table 4:** Analysis of adult survival using a regression for a parametric survival model. (A) Global: with the effect of habitat, adult body size, rearing season. Then per habitat: (B) Permanent habitat, and (C) temporary habitat: with the effect of adult body size and rearing season. *P*-values in bold are significant at the 0.05 level

Source	Value	Standard error	*P*
**(A) Global**			
Habitat	−4.395	2.028	**0.003**
Size	0.284	0.258	**0.027**
Spring season	0.482	0.178	**0.006**
Temporary habitat × size	1.233	0.511	**0.016**
Temporary habitat × spring season	0.351	0.285	0.218
Log(scale)	−0.334	0.075	**< 0.001**
**(B) Temporary habitat**			
Size	1.593	0.501	**< 0.001**
Spring season	0.892	0.258	**0.001**
Log(scale)	−0.189	0.122	0.123
**(C) Permanent habitat**			
Size	0.325	0.235	0.167
Spring season	−0.003	0.163	**0.008**
Log(scale)	−0.425	0.093	**< 0.001**

### Season and size effect

For juveniles, we detected no survival difference between individuals reared in fall and those reared during spring (Fig. 2B, Table 2); and only a small difference with the nonparametric analysis (data not shown). Conversely, we found a difference in survival between big and small individuals, using the median shell size to separate between those two groups (Fig. 2C, log rank test on survival curve between sizes *P* < 0.05). Body size had thus an important positive effect on juveniles’ survival (Fig. 2C, Table 2). In adults, we found a significant effect of the rearing season(Fig. 3B and Table 4A) and body size (Fig. 3C and Table 4A) on survival: big adults reared in spring survive better than others (confirmed also by log rank test on survival curves between seasons and sizes, for both *P* < 0.01). However, the parametric analysis in Table 4A (but also suspicion on Fig. 3B and 3C) showed significant interactions between habitat and season and between habitat and body size. Since the interactions between variables might render the simple effects difficult to interpret, we analyzed data from each habitat (temporary and permanent) separately (Tables 4B and 4C). In temporary habitat, significant effect of adult size was detected: survival increases with body size (Table 4B). The scale parameter in this model does not significantly differ from one (scale = 0.806, *P* value = 3.7e^–5^) indicating that mortality rate in the experiment was constant through time for individuals from temporary habitat (Table 4B). Conversely, for permanent habitat, we detected a marginal effect of adult season and no effect of adult size on survival (Table 4C) while the scale parameter was significantly lower than one (scale = 0.665, *P* value = 0.031). This indicates that during the experiment mortality rate decreased over time for individuals from permanent habitat (Table 4C).

**Table 2:** Analysis of juvenile survival using a regression for a parametric survival model with the effect of juvenile body size and the parents' season of rearing. *P*-values in bold are significant at the 0.05 level

Source	Value	Df	Deviance	*P*
Temporary habitat	−0.014	1	0.030	0.862
Size	1.383	1	53.567	**< 0.001**
Spring season	0.149	1	3.314	0.070
Log(scale)	−0.979	1	84.381	**< 0.001**

## Discussion

This study provides a new experimental evidence of selection and local adaptation phenomena in temporary waters in a freshwater species. Indeed, our results show that in the freshwater snail *Galba truncatula*, survival to artificial drought in laboratory conditions differs between permanent and temporary habitats of origin and also between life stages. Adults from temporary habitats were found to be more resistant to desiccation than adults from permanent ones, while no such difference was detected in juveniles. We will first discuss the proximal mechanisms of that resistance. We will then conclude on local adaptation and on life history evolution in heterogeneous environment.

For adults, this study first sheds some light on the mechanisms that may underlie the observed differences among habitats in resistance to drought. *Galba truncatula* is a nonoperculate pulmonate snail, and consequently, the aperture cannot be closed to prevent water loss. However, adults have been reported to survive to drought conditions for several weeks or even months (Peters 1938; Kendall 1949); several factors have been proposed to explain such resistance. In natural conditions, snails may stick their aperture to the soil surface (thus reducing dehydration), or hide into the deeper ground where humidity can be conserved (Kendall 1949). However, in our experiment with Petri dishes, the latter point at least could not have had any influence on resistance differences. We can thus focus on two physical factors. First, increased shell size can be a mechanism of desiccation resistance: larger size is known to reduce water loss by conferring a better surface-volume ratio (Dudgeon 1981; Facon et al. 2003). This is confirmed by the present study, as size was found to positively correlate with survival to desiccation for adults. Second, the rearing season seems to have an effect on survival, we observed that “old” individuals survive less than “young” ones. However, after correcting for both, individuals from temporary habitats still show a better survival to desiccation than ones from permanent habitat, suggesting that another factor is involved in resistance. The nature of this hidden factor cannot be determined here and, for instance, in addition to shell size, physiological differences could explain the different resistance patterns between habitats. Such physiological mechanisms have been found, for example, in *Drosophila melanogaster*, where increased glycogen reserves enhance desiccation resistance (Graves et al. 1992; Gibbs et al. 1997, 2003). Per habitat, the effect of this hidden variable can also be observed. In our experiment, individuals that do not resist to desiccation die first. If individuals differ in their resistance to drought, thus, average death rate should decrease during the course of the experiment. In temporary habitat, the scale parameter that quantifies death rate variation over time equals one: once differences in survival due to differences in size or rearing season are controlled in our statistical model, we do not detect any additional difference in resistance between individuals. In permanent habitat, conversely, the estimated scale is lower than one. This indicates that there are differences in survival between individuals, which cannot be explained by differences in size or season. Said differently, in both habitats survival depends on size (and rearing season) but in permanent habitat there must be some hidden factor that varies among individuals and creates differences in resistance to drought.

Beyond finding several factors correlated to survival, our study provides direct evidence for local adaptation in a highly heterogeneous natural environment (in terms of desiccation risks) at a small geographical scale. One can say that other scenarios than local adaptation can lead to the same pattern. First, geography can be a confounding factor but here populations from different habitats can be in the same locality and the random geographical distribution of habitats was already confirmed (Chapuis et al. 2007). We observe also no correlation between size and geographic distances between populations (Mantel test, data not shown). Second, genetic drift due to successive drought or colonization bottlenecks could be the cause of a reduction in genetic variance (see Pujol and Pannell 2008) and thus explain differences observed among habitats. However, this alternative explanation can be rejected because populations in temporary habitats show no significant difference in neutral diversity (*H*E and *R*S, the expected heterozigosity and the allelic richness, respectively) than permanent populations as we found previously (Chapuis et al. 2007). Additionally, the effective population size was found not different among habitats in a previous study on *Galba truncatula* (Trouvé et al. 2005). Those points suggest that temporary populations are not more subject to bottlenecks that permanent ones and thus this could not affect the quantitative genetic variation in temporary populations. Finally, variation in the mating system between habitats could have an effect but the selfing rate does not differ among habitats (Chapuis et al. 2007). By consequence, we can conclude that local adaptation is the most likely scenario related to the variation in life history traits observed here.

Overall, our study might explain some of the “surprising” life history trait values we previously documented in *Galba truncatula* (Chapuis et al. 2007). Indeed, individuals from permanent habitat mature at a younger age, at a smaller size, and lay more eggs in 30 days than individuals from temporary habitat. It may be “surprising” because, according to the theory of McArthur and Wilson (1967), we expected to observe the opposite pattern: individuals living in temporary habitat (and therefore unpredictable) to sexually mature earlier, to have a smaller size and to be more fecund than individuals from permanent habitat (stable). However, we found here that a big size helps survive to drought periods and thus is certainly selected in temporary habitats despite our first expectation. Drought selection may also act on other traits directly or indirectly (by trade-offs between traits). For instance, we also observed previously that individuals from temporary habitat have a smaller fecundity than individuals from permanent habitat (Chapuis et al. 2007). This pattern can be interpreted as a cost of resistance in genotypes from the temporary habitat. This late reproduction may be the cost to pay for producing individuals that (1) are larger (and thus more resistant) and (2) show increased resistance to desiccation.
